# A Polynucleotide Repeat Expansion Causing Temperature-Sensitivity Persists in Wild Irish Accessions of *Arabidopsis thaliana*

**DOI:** 10.3389/fpls.2016.01311

**Published:** 2016-08-31

**Authors:** Amanda Tabib, Sailaja Vishwanathan, Andrei Seleznev, Peter C. McKeown, Tim Downing, Craig Dent, Eduardo Sanchez-Bermejo, Luana Colling, Charles Spillane, Sureshkumar Balasubramanian

**Affiliations:** ^1^School of Biological Sciences, Monash UniversityMelbourne, VIC, Australia; ^2^Genetics and Biotechnology Laboratory, Plant and AgriBiosciences Research Centre, School of Natural Sciences, National University of IrelandGalway, Ireland; ^3^School of Biotechnology, Dublin City UniversityDublin, Ireland

**Keywords:** triplet expansion, cryptic genetic variation, natural variation, ambient temperature, QTL analysis, genetic modifier, polynucleotide repeat

## Abstract

Triplet repeat expansions underlie several human genetic diseases such as Huntington's disease and Friedreich's ataxia. Although such mutations are primarily known from humans, a triplet expansion associated genetic defect has also been reported at the *IIL1* locus in the Bur-0 accession of the model plant *Arabidopsis thaliana.* The *IIL1* triplet expansion is an example of cryptic genetic variation as its phenotypic effects are seen only under genetic or environmental perturbation, with high temperatures resulting in a growth defect. Here we demonstrate that the *IIL1* triplet expansion associated growth defect is not a general stress response and is specific to particular environmental perturbations. We also confirm and map genetic modifiers that suppress the effect of *IIL1* triplet repeat expansion. By collecting and analyzing accessions from the island of Ireland, we recover the repeat expansion in wild populations suggesting that the repeat expansion has persisted at least 60 years in Ireland. Through genome-wide genotyping, we show that the repeat expansion is present in diverse Irish populations. Our findings indicate that even deleterious alleles can persist in populations if their effect is conditional. Our study demonstrates that analysis of groups of wild populations is a powerful tool for understanding the dynamics of cryptic genetic variation.

## Introduction

Allelic differences in simple polynucleotide repeats are associated with phenotypic variation in several species (Verstrepen et al., 2005; Levdansky et al., 2007; Michael et al., 2007; Vinces et al., 2009). In extreme cases, this variation can lead to genetic diseases, as exemplified by the triplet expansion diseases in humans. Only one example of a triplet repeat expansion-associated phenotype is known outside of humans, the *irregularly impaired leaves* (*iil*) phenotype, which was discovered in the Burren-0 (Bur-0) accession of *Arabidopsis thaliana* (L.) Heynh. (Brassicaceae; hereafter *A. thaliana*) (Sureshkumar et al., 2009). The *iil* phenotype occurs due to a dramatic expansion of a GAA/TTC trinucleotide repeat in the third intron of the *ISOPROPYL MALATE ISOMERASE LARGE SUBUNIT 1* (*IIL1*, At4g13430) gene. The trinucleotide repeat expansion, when present in a homozygous state, causes a temperature-dependent reduction in *IIL1* transcript levels and severely impairs growth of this accession under short days at elevated temperatures (Sureshkumar et al., 2009).

The *IIL1* locus was mapped and cloned using an F_2_ population derived from the Bur-0 × Pf-0, in which the *iil* phenotype segregated as a large effect locus. However, in a Bur-0 × Col-0 F_2_, such segregation could not be observed indicating the presence of genetic modifiers (Sureshkumar et al., 2009). Furthermore, the *iil* phenotype could be observed only upon elevated temperatures (27°C, short days) and not in standard growth conditions (23°C, long days). Thus, the phenotypic impact of the repeat expansion could be seen only upon genetic or environmental perturbation, indicating that this repeat expansion represents a cryptic genetic variation (Paaby and Rockman, 2014). There is considerable interest in the analysis of cryptic genetic variation, as it can represent either adaptive potential or a source of deleterious alleles that require constant suppression. Such hidden genetic substrates could become important upon a change in the environment and may play a critical role in shaping evolution (Gibson and Dworkin, 2004; Ledon-Rettig et al., 2014; Paaby and Rockman, 2014). The strong deleterious nature of the *IIL1* triplet repeat expansion is consistent with our previous findings that the GAA/TTC triplet repeat expansion is rare, and may be unique to Bur-0.

Bur-0 is the only accession of *A. thaliana* collected from Ireland, which is available from stock centers and part of the 1001 genome project (Alonso-Blanco et al., 2016). Bur-0 was originally collected more than 50 years ago (1959) in the Burren region of County Clare and County Galway in western Ireland. The Burren is a region of karstified limestone with unique botanical features (McNamara and Hennessy, 2010). The Burren region supports an unusual array of Mediterranean and Arctic-Alpine plants growing side by side at close to sea level (Webb, 1961; Webb and Scannell, 1983). Given the cryptic nature of the *iil* phenotype we considered whether the GAA/TTC repeat expansion might still persist in the wild. Regional populations of *A. thaliana* have been shown to be complementary to the global collection of accessions particularly for analyzing forces that shape evolution at local levels (Le Corre et al., 2002; Pico et al., 2008; Bomblies et al., 2010; Long et al., 2013; Méndez-Vigo et al., 2015; Sasaki et al., 2015). For instance, while the analysis of global populations has revealed common allelic variation contributing to flowering time differences, analysis of local populations has uncovered selection operating at a local level for early flowering through an excess of non-synonymous divergence polymorphisms (Johanson et al., 2000; Le Corre et al., 2002; Méndez-Vigo et al., 2015). Therefore, we reasoned that the analysis of Irish populations would allow us to assess whether the GAA/TTC repeat expansion still persists, and whether it is unique to the environs of the Burren region.

In this paper, we demonstrate that the *IIL1* repeat expansion is a cryptic genetic variation that is revealed only under specific environmental conditions such as high temperature and UV-B exposure. We also demonstrate that genetic modifiers can mask the *iil* phenotype and present Quantitative Trait Locus (QTL) mapping for one of these modifiers. Furthermore, we collect and analyze *A. thaliana* accessions and demonstrate that the *IIL1* triplet has persisted in multiple independent natural populations for over 50 years. Our results significantly expand our understanding of the persistence of cryptic genetic variation within plant species.

## Materials and methods

### Plant growth, DNA extraction, and sequencing

The Bur-0 × Col-0 recombinant inbred lines (RILs) have been described previously (Simon et al., 2008). The collection of wild accessions from Ireland is described below. Plants were grown either in long days (LD, 16 h light/8 h dark) at 23°C for seed collection and in short days (SD, 8 h light/16 h dark) at 23°C or 27°C for *iil* phenotyping, QTL analysis and temperature assays as described previously (Sanchez-Bermejo et al., 2015). For Diversity Arrays Technology sequencing (DArT-seq), DNA from the plant from which the seeds were bulked was extracted as described previously (Balasubramanian et al., 2006). GAA/TTC repeat expansion was analyzed through Polymerase chain reaction (PCR) using primers described in Table S1. For sequencing the microsatellite repeats, purified PCR products were sequenced (Micromon, Monash University) and analyzed through Seqman (DNAStar-Lasergene).

### Stress assays

Fifty seeds of the accession Bur-0 and a natural suppressor in the Bur-0 background were grown under different environmental perturbations. These included drought, salt stress, osmotic stress, abscisic acid treatment, oxidative stress, UV-B exposure, wounding, pH variation and high temperature. Previously published treatment methods were used for each of the stress assays (Xing and Rajashekar, 2001; Xiong and Zhu, 2002; Kilian et al., 2007; Walley et al., 2007; Huang et al., 2008; Galpaz and Reymond, 2010; Fujita et al., 2012). Each assay is briefly described below. For drought assays, watering was stopped from 2-weeks after germination until the soil was dry (Huang et al., 2008). Salt stress was assayed at 150 mM sodium chloride concentration either in plates or by pouring 100 mL of the solution on the soil surface and then allowing the same to dry (Galpaz and Reymond, 2010). Similarly for osmotic stress 400 mM mannitol solution was poured over the soil. Plants were also assayed in plates containing 300 mM mannitol (Xiong and Zhu, 2002). ABA treatment was done by spraying, as described previously (Xing and Rajashekar, 2001). For oxidative stress, plants were assayed in MS plates containing 10 or 0.3 uM methyl viologen (Fujita et al., 2012). By incising rosette leaves using a forceps, wounding was carried out (Walley et al., 2007). For UV-B treatment, 2-week old plants were irradiated for 5–10 min with UV-B light with a biologically effective quantity of 1.18 W m^−2^ (Kilian et al., 2007). The plants were then transferred back to the growth chambers and the phenotypes were assayed after 2 weeks. To vary the pH sodium bicarbonate was added to MS plates making the plates slightly alkaline.

### QTL analysis of the modifier locus

To analyze germination and flowering time, six bulked seeds per accession were sown in a fully randomized design in individually numbered positions of multiple-well soil flats. For phenotyping, eight plants of each of the 344 RILs were grown in a growth room at 27°C in short days. Plants were grown as single individuals in 30-well trays in a completely randomized design as described previously (Lempe et al., 2005). The trays were repositioned systematically on a weekly basis to minimize the effect of micro-environmental variation. The *iil* phenotype was first assessed against the parental Bur-0 line. We first photographed all the variant phenotypes and generated a scale of 1–10 (Figure S2). Using this scale, every plant was scored and a quantitative score for the *iil* phenotype was obtained (Table S2). Flowering time was measured as Days To Flower (DTF), as described previously (Sanchez-Bermejo et al., 2015). QTL mapping was done using R/qtl package through simple interval mapping or two-dimensional scans (Broman et al., 2003).

### Collection of arabidopsis accessions from Ireland

Plants and seeds of *A. thaliana* plants growing wild were collected from the island of Ireland. Our first collection was made from the Burren region from which the Bur-0 strain is reported to have been originally collected (http://www.arabidopsis.org). This region was defined as the areas of north Co. Clare and adjacent parts of southeast Co. Galway associated with visible karstified limestone. Wild plants were sourced from all roadsides, field-sides, churchyards and graveyards, accessible open pastures, and other places suitable for the growth of weedy ruderals. Our second set of collections comprised additional accessions from other areas of the island of Ireland, chosen from recent records of *A. thaliana* obtained from the Botanical Society of Britain and Ireland (BSBI; http://www.brc.ac.uk/plantatlas/index.php?q=node/2250). In each case, multiple individuals were collected from the same stand, with seed from 5 to 10 plants sampled from each wherever available. The entire Irish *Arabidopsis thaliana* collection, consisting of 529 samples that represent 150 locations is listed in Table S3. Seeds from plants representing different stands were first grown and we bulked the seeds by growing plants at 23°C long days. After bulking, we consolidated a collection of 131 accessions (127 accessions and 4 controls (Bur-0, NS10, NS15, and Col-0) and this set was used in further analyses (Table S4).

### Phenotyping of the Irish arabidopsis accessions

Seeds were stratified for 4 days in the dark at 4°C then moved to different growth conditions depending on the experiment. For plate assays (hypocotyl elongation/root length), seeds were sterilized in 1.5 ml tubes in 70% ethanol with 0.1% Triton X-100 for 3–5 min and washed in 95% ethanol for 1–2 min. Seeds were then spotted onto 0.5 × MS media plates. Root length was measured 5 days post-germination and hypocotyl elongation measured 10 days post-germination from photographic images, using ImageJ64 software for Macintosh (National Institutes of Health, Bethesda, Maryland, USA). Temperature sensitivities in root length and hypocotyl elongation among wild accessions were calculated from regression of hypocotyl/root length onto temperature means (23SD vs. 27SD). The impact of repeat expansion, soil type or the genotype on phenotypes as well as genotype × environment interactions were analyzed using JMP (SAS Institute, USA) through appropriate analysis of variance (ANOVA) models. For example, the effect of repeat expansion was assessed using a model with the repeat expansion as the factor and hypocotyl length or temperature sensitivity as a response. Degree of leaf serration was based on a relative scale generated from within the dataset ranging from 1 to 9 (Figure S12). A single representative image of each of the 131 accessions was taken of 4-week old plants and three independent volunteers were asked to score the degree of serration of all rosette leaves, and the mean serration value calculated for each accession.

### Genotyping of the Irish arabidopsis accessions

Genotyping of 131 plants, which were bulked, was performed by Diversity Arrays Technology Pty Ltd (DArT P/L, Canberra, Australia) as described previously (Sansaloni et al., 2010). Samples with >10% missing data were excluded. Minor allele frequency (MAF) was set to 0.05% and all taxa markers missing data in over 20% of loci were excluded from the analysis. The selected DArT markers had Call Rate (percentage of samples that could be scored as “0” or “1”) > 80%, Reproducibility (reproducibility of scoring between replicated samples) > 97% and were polymorphic with frequencies of samples scored as “0” or “1” ranging between 0.95 and 0.05. We also ensured that the genotypic data contained SNPs that are uniquely mapping. This filtering resulted in a dataset of 4556 SNP markers for a total of 131 accessions, which was then used in further analyses. The repeat number was quantified for each accession that lacked the expansion (Table S4). For the strains containing the repeat expansion (more than 200+ repeats), the copy number of the repeats was estimated based on the molecular size of PCR amplicons.

### Analysis of population structure of Irish arabidopsis accessions

Population structure was analyzed using the model-based Bayesian clustering algorithm implemented by STRUCTURE v.2.3.4 (Pritchard et al., 2000) and the kinship procedure of Hardy (Hardy, 2003) using SPAGeDi (Hardy and Vekemans, 2002), respectively. STRUCTURE analyses were performed assuming an admixture model with default settings (no informative priors were used). STRUCTURE was run from 1 to 10 inferred clusters (*K*) with 4 independent runs for each *K*, each run starting with a burn-in period of 100,000 steps followed by 100,000 Markov Chain Monte Carlo iterations. The most probable value of *K* was selected according to the Evanno method (Evanno et al., 2005), implemented through the Structure Harvester web tool (Earl and vonHoldt, 2012). To assess population structure at the chromosomal level we calculated genetic distances to construct genome-wide and chromosome unrooted neighbor-joining trees from maximum-likelihood distances inferred with a TN93 substitution model constructed for all using Perl scripts and the package Ape v3.3 (Paradis et al., 2004) in RStudio v0.00.467 for 4556 uniquely mapping non-duplicate sites.

## Results

### High temperatures and UV-B exposure unmask cryptic genetic variation in Bur-0

**Figure 1:**
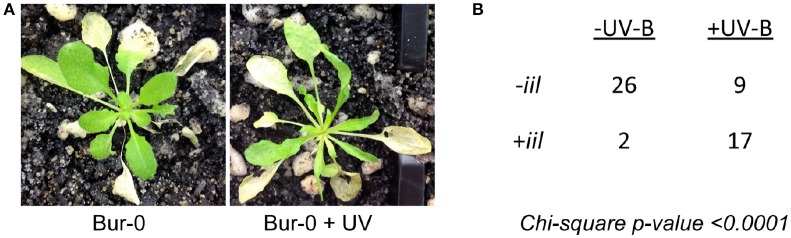
**UV-B can induce the *iil* phenotype even at lower temperature conditions in the Bur-0 accession**. **(A)** Bur-0 plants growing at 23°C either with or without UV exposure. **(B)** The number of plants that display the *iil* phenotype with (+UV-B) or without (−UV-B).

The *iil* phenotype, caused by the GAA/TTC triplet expansion, is observed only under elevated temperatures in Bur-0 (Sureshkumar et al., 2009). To investigate whether the *iil* phenotype is a general stress response, we subjected the Bur-0 accession to multiple stresses at lower temperature (23°C) and assessed for the appearance of the *iil* phenotype. Among the tested conditions (drought, salinity, osmotic stress, mannitol treatment, abscisic acid treatment, oxidative stress, wounding, nutrient deprivation, alkalinity stress, UV-B exposure), we did not observe the *iil* phenotype in any stress condition except for the UV-B exposure (Figure 1A). At 23°C short days in our conditions, we observed 2 out of 28 Bur-0 plants displaying any resemblance to the *iil* phenotype. In contrast, we observed 17 out of 26 UV-B exposed plants displaying the *iil* phenotype at 23°C (Figure 1B). Analysis of the repeat expansion in these plants revealed that the UV-B exposed plants displayed higher level of variability in the repeat expansion compared to the un-treated plants (Figure S1). Therefore, the appearance of the *iil* phenotype does not appear to be a general stress response, but somewhat specific to temperature (high temperature) and light (UV-B) alterations.

### Genetic modifiers mask the *iil* phenotype in varied genetic backgrounds

**Figure 2:**
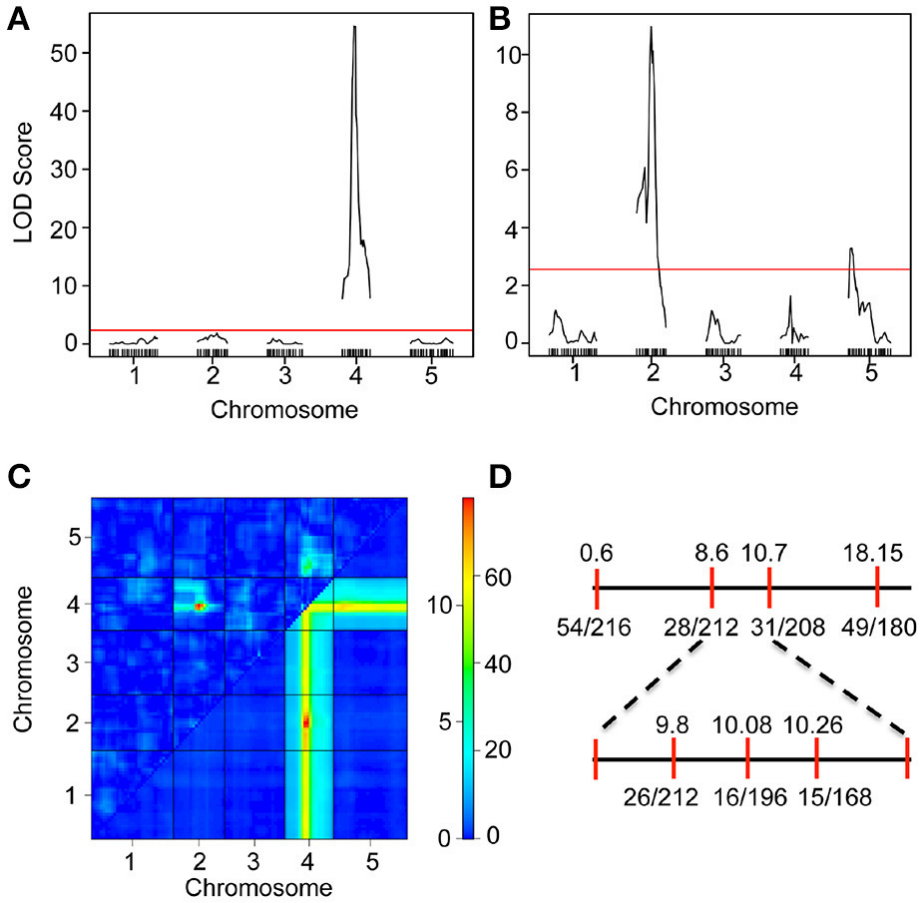
**Mapping of a modifier of *iil1* in the Col-0 × Bur-0 recombinant inbred lines. (A)** QTL map of the *iil* phenotype in the Bur-0 × Col-0 RIL population. **(B)** QTL analysis of the modifier using RILs, which harbor the Bur-0 allele in Chromosome 4. The red lines in **(A,B)** represent LOD thresholds determined through 1000 permutations. **(C)** Two-dimensional scan of the QTLs representing genetic interactions between the loci. The top triangle represents epistatic interactions. The bottom triangle represents additive interaction. The color scale bar represents LOD scores for epistatic (scale 0 to >10) and additive (scale 0 to >60) interactions. The QTL on chromosome 2 exhibits both additive and epistatic interactions. **(D)** Linkage analysis of the *iil* phenotype in Bur-0 × Col-0 F_2_ population.

We have previously shown that the *iil* phenotype segregates in a Mendelian manner only in certain genetic crosses (e.g., Bur-0 × Pf-0) but not in others (e.g., Bur-0 × Col-0), which suggested the presence of genetic modifiers that can mask the phenotypic impact of the GAA/TTC repeat expansion (Sureshkumar et al., 2009). To investigate the genetic architecture of the modifier(s), we analyzed RILs derived from Bur-0 × Col-0 cross. A total of 2752 plants belonging to 344 RILs (8 plants/line) were quantitatively scored (Figure S2 and Table S2) for their *iil* phenotype at 27°C in short days. Using this quantitative phenotypic score, we mapped the QTLs controlling variation in the *iil* phenotype. A single QTL with a very high LOD score was detected at chromosome 4 co-localizing with the GAA/TTC repeat expansion, consistent with previous findings (Figure 2A, Sureshkumar et al., 2009). To assess other loci that influence variation in the *iil* phenotype, we selected lines that are homozygous across the GAA/TTC repeat expansion and performed QTL analysis. Through this analysis, we detected the modifier QTL to be present in the 8–10 Mb region of chromosome 2 (Figure 2B). Another modifier locus was detected at the top of chromosome 5 (Figure 2B). Two-dimensional scans revealed that both modifier loci exhibit epistatic interaction with the QTL localizing to the repeat expansion (Figure 2C). However, the modifier on chromosome 2 also exhibited an additive effect with the QTL localizing to the repeat expansion (Figure 2C). RILs that harbor a Col-0 allele at this locus appear to suppress the effect of the repeat expansion at the phenotypic level. Thus, complex genetic interactions modify the appearance of the *iil* phenotype in the Bur-0 × Col-0 RIL population. To confirm this QTL, we analyzed another set of F2 populations derived from a cross between Bur-0 × Col-0. Using plants that displayed the *iil* phenotype in the F2 population, we confirmed the linkage to chromosome 2 (Figure 2D). Although, the observed linkage reflects the underlying genetic complexity, the modifier locus was mapped between 8.6 and 10.7 Mb on chromosome 2 (Figure 2D).

### Recovery of the *IIL1* repeat-expansion in wild Irish accessions of arabidopsis

**Figure 3:**
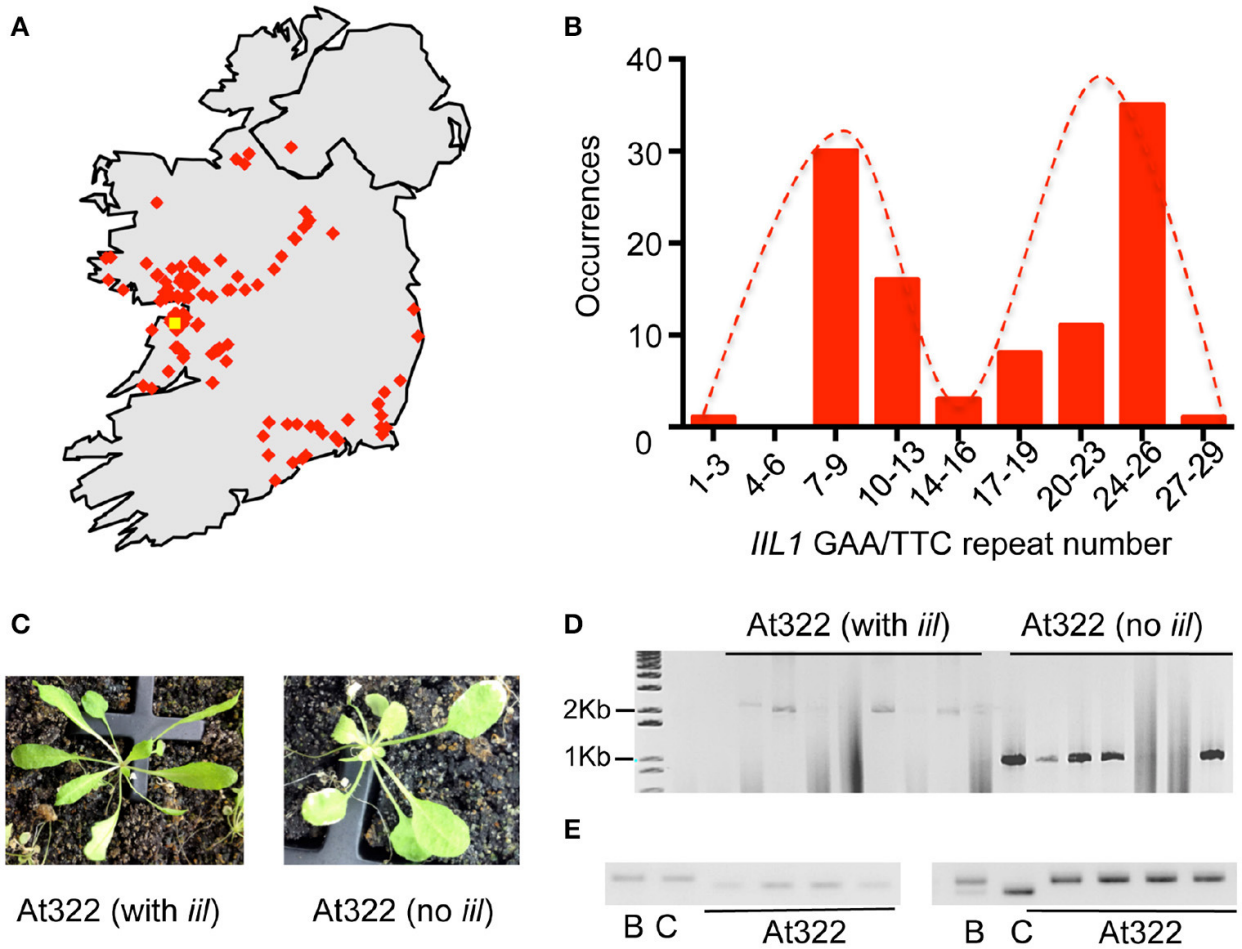
**Recovery of the *IIL1* repeat expansion in wild accessions from Ireland. (A)** The geographic locations of accessions collected from Ireland. The yellow dot indicates the possible location at which the original Bur-0 accession was collected in 1958; red dots indicate approximate locations of wild accessions collected in 2011/2012. **(B)** Distribution of the *IIL1* GAA/TTC repeat-numbers in wild accessions. The distribution is restricted to those accessions that do not harbor the expansion. **(C)** The *iil* phenotype recovered in the segregating progeny of plants of collected line At322 along with plants that lacked the *iil* phenotype. **(D)** PCR analysis of repeat length on segregating progeny of At322 with and without the *iil* phenotype. **(E)** PCR analysis for two SSLP markers on At322. B- Bur-0, C-Col-0, At322—four plants from the segregating progeny of the wild accession At322 that display the *iil* phenotype. The two markers are LUGSSLP531 (left) and MSat2.36 (right).

Since our studies with the Bur-0 accession indicated that both genetic and environmental factors could mask the *iil* phenotype, we reasoned that the *IIL1* repeat expansion may still persist in wild Irish Arabidopsis populations. Bur-0, the only available accession of *A. thaliana* from Ireland is reported to have been collected near a wall by a roadside in the Burren, Co. Clare, Ireland by Albert Kranz in 1958. To assess the persistence of the repeat expansion, two targeted collections of *A. thaliana* were made from populations in Ireland during the springs and summers of 2011 and 2012. The first collection was targeted on the region of the Burren itself, the second from other parts of Ireland (Figure 3A, Table S3). In their natural habitats plants exhibited phenotypic differences, for example in leaf morphology and plant stature (Figure S3). The seeds were collected and plants were re-grown in the lab to generate single-seed descendent plants, which resulted in a consolidated collection of 131 accessions representing different locations across Ireland (Table S4).

To determine whether the *IIL1* triplet repeat expansion still persists in Irish populations, we analyzed the accessions collected from Ireland through PCR analysis for the *IIL1* expansion, phenotyping at 27°C in short days and sequencing. Most of the accessions did not contain the *IIL1* repeat expansion as expected. Sequencing of the repeat region revealed that the *IIL1* GAA/TTC repeat length in the accessions varied from 3 to 28 repeats. This range of repeat length is similar to the 0–36 repeats seen in global populations previously (Sureshkumar et al., 2009). However, the distribution of the repeats revealed a bimodal distribution with a prevalence of 9 and 26 repeats in a majority of accessions, which differs from what is seen in global populations (Figure 3B).

Through this analysis we identified a total of 10 accessions (At216, At249, At319, At322, At369, At370, At379, At434, At460-462, and At533) from seven geographic locations that contained the *IIL1* repeat expansion along with the typical *iil* phenotype at 27°C short days (Figure 3C, Figure S4). In some accessions, not all plants displayed the *iil* phenotype (Figure 3C). Nevertheless, there was a clear correlation between the presence of the *iil* phenotype and the repeat expansion, while the plants that lacked the *iil* phenotype typically harbored shorter repeats. This suggests that some of the 10 original accessions were heterozygous for the repeat expansion (Figure 3D). To ensure that the accessions we recovered in the wild do not represent contamination from seeds of the Bur-0 lab accession, we genotyped At322 with 23 SSLP markers distributed across the genome. This analysis confirmed that At322 is distinct from Bur-0 as well as Col-0 (Figure 3E, Table S5). Thus, we have recovered the GAA/TTC expansion at the *IIL1* locus, from at least 10 distinct wild *A. thaliana* stands in Ireland out of a total of 131 samples.

### Expanded repeats occur in genetically diverse Irish *A. thaliana* populations

To determine whether the accessions that carry the repeat expansion are genetically similar to each other, we analyzed the genetic diversity among Irish accessions using DArT-seq (Raman et al., 2015). From the DArT-Seq data, we compiled data for 4556 SNPs for which genotypic information was available for over 90% of the accessions and utilized this data to analyze the genetic architecture of the samples that we collected in Ireland (Table S6).

**Figure 4:**
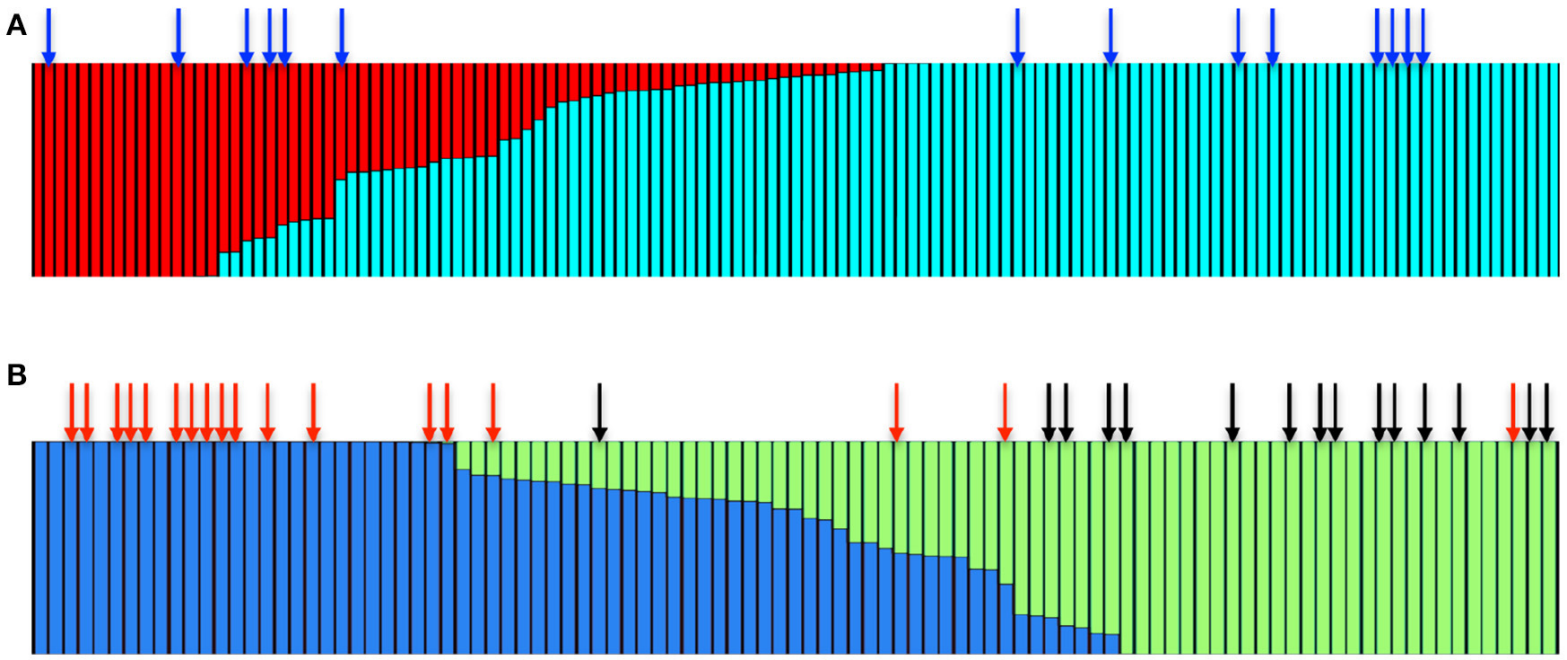
**The *IIL1* triplet repeat expansion is present in genetically diverse accessions in Ireland. (A)** STRUCTURE bar plot colors show the fraction of ancestry assigned to each of the *K* = 2 populations. The blue arrow represents the accessions that harbor the repeat expansion **(B)** Bar plot of the major sub-cluster of accessions (shown in blue in **A**) fall into *K* = 2 groups. Red and black arrows represent accessions that harbor either 9 or 26 GAA/TTC repeats at the third intron of the *IIL1* locus.

STRUCTURE analysis (Pritchard et al., 2000) using the admixture model for multilocus genotype data suggested that the wild Irish *A. thaliana* populations consist of two main clusters (*K* = 2, Figure 4A). The majority of accessions were primarily associated with one cluster, while the other cluster was relatively small. We therefore analyzed only the subset of the accessions that fell in the major cluster through STRUCTURE to decipher whether there are further sub-clades. This analysis identified that the major cluster itself consisted of two sub-clusters (*K* = 2, Figure 4B, Figure S5). To assess whether the accessions that harbor the repeat expansion are confined to one of the sub-clusters, we mapped the distinct accessions on to the STRUCTURE Harvester output (Earl and vonHoldt, 2012). In the non-expanded range, the bimodal distribution of the repeat number was reflected in the STRUCTURE analysis. The accessions that harbored 26 repeats predominantly fell in one sub-cluster and the accessions that carried 9 repeats fell in another sub-cluster (Figure 4B). We could detect that the accessions carrying the *IIL1* repeat expansion in both sub clusters (Figure 4A), suggesting that the repeat expansion is present in diverse genetic backgrounds. Chromosomal level analysis also confirmed the same (Figures S6–S11), which suggests that population structure alone cannot explain the observed genetic patterns; and that the expansion either predates the sub-division of the Irish accessions of Arabidopsis or has spread through the population after originating in one of the sub-populations.

### Changes in triplet repeat length is associated with phenotypic variability

**Figure 5:**
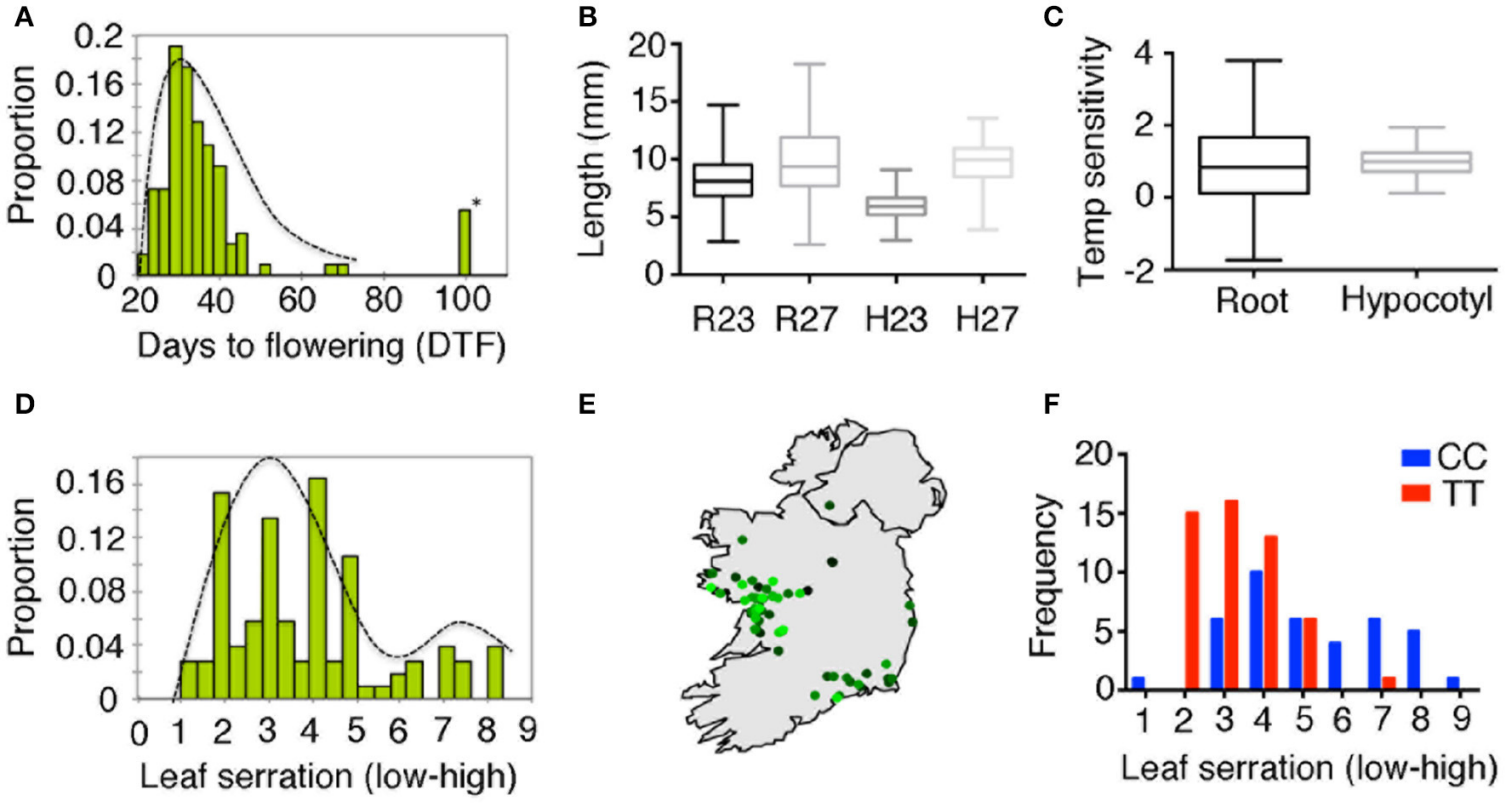
**Phenotypic variation in wild accessions from Ireland**. **(A)** Distribution of flowering time expressed as days to flowering in 23°C in long days. Asterisk indicates accessions with extremely later flowering (>5 months, presumed to be winter annuals). **(B)** Distribution of root length (R23, R27) and hypocotyl length (H23 and H27) at 23 and 27°C in short days shown as box plots. **(C)** Box-plots for temperature sensitivity in root and hypocotyl length. **(D)** Distribution of leaf serration among 131 Irish accessions. **(E)** Geographic distribution of leaf serration across Ireland, where light green to dark green represents low to high serration. **(F)** Differences in the distribution for leaf serration among Irish accessions correlated with the genotype at 7.85 Mb of chromosome 4 (DArT-seq marker ID: 100017906).

As the *iil* phenotype caused by triplet expansion is visible only under high temperature or UV-B stress, it is likely that this phenotype is not commonly manifest in the wild under Irish growth conditions. To investigate whether the repeat expansion is associated with any other phenotypes, we undertook a phenotypic analysis of our Irish collection root length, hypocotyl elongation, degree of leaf serration and flowering time. Since the *iil* phenotype is temperature-sensitive, we also measured temperature sensitivity root and hypocotyl length (Figures 5A–D). Considerable genetically controlled phenotypic variation was found (Table S7). ANOVA analysis with repeat expansion (i.e., whether the repeat is normal range or expanded), detected a significant association between the repeat expansion and hypocotyl length at 27°C, which accounted for 13% of the variation (*p* < 0.001), which was also reflected in temperature sensitivity (*p* < 0.01) (Figures S13A,B, Table S4). QTL analysis of flowering time at 27°C revealed a minor QTL spanning the repeat expansion (Figure S13C). In addition, we observed the natural suppressor NS15 (NS15 lacks the repeat expansion in an otherwise Bur-0 background) flowered earlier in long days (Figure S13D). Thus, plants with the repeat expansion appeared less sensitive to temperature changes in hypocotyl length, and potentially flowering time as well, which suggests that the expanded repeats can have additional phenotypic consequences.

Variation in triplet repeat length, even in their non-expanded ranges, has been shown to be associated with phenotypic differences in other species (Levdansky et al., 2007; Michael et al., 2007). To assess the impact of the repeat length in the non-expanded range, we explored a model with different phenotypes as response and repeat number as a factor, but excluding accessions with the expansions (Table S4). While we were unable to detect an impact on flowering time, we observed significant associations between the copy number of the repeats and the degree of leaf serration (*p* < 0.02, 8% of variation), temperature sensitivity in root length (*p* < 0.01, 10% of variation) and hypocotyl elongation (*p* < 0.01, 13% of variation). In addition, we observed an impact of soil type on root length at both temperatures [*p* < 0.0008 (23°C) and *p* < 0.001 (27°C), 20% variation] and leaf serration (*p* < 0.0001, 26% of variation) consistent with the observed geographic pattern (Figure 5E). Furthermore, we observed that one of the markers (Marker ID:100017906) at position 7.85 Mb was strongly associated with leaf serration (Figure 5F, *p* < 0.0001). However, with the major groups of repeats (9 or 26 repeats) mapping to specific sub-clusters, this could also be explained by a strong effect of population structure. Thus, geographic differentiation and population structure accounts for most of the variation in leaf serration in the Irish accessions.

## Discussion

### UV-B and high temperature unmasks the *iil* phenotype caused by triplet repeat variation

We have demonstrated that the *iil* phenotype is not a generic stress associated phenotype but is instead specific to certain types of environmental modulations. We have shown that plants grown at 23°C have a propensity to display the *iil* phenotype associated with the repeat expansion when exposed to UV-B. The association between UV-B exposure and the *iil* phenotype was statistically significant (*p* < 0.0001, chi-square test) and was correlated with an increase in the instability of the repeat expansion. UV-B exposure is known to have many effects on plant growth and development such as decreased plant height, decreased leaf area, reduced stomata number and curling of leaves, as well as an increase in axillary branching (Jansen et al., 1998). Indeed leaf development is one of the key phenotypes that can be affected through UV-B exposure. In addition, it is also known that the Arabidopsis photolyase, which is induced upon UV-B exposure and is responsible for the photo reactivation mechanisms is also temperature sensitive (Pang and Hays, 1991). Our analysis of *IIL1* repeats revealed that upon UV-B exposure, there was an increased propensity to expand associated with increased instability (Figure S1). This is consistent with the increase in the number of *iil* plants and it is possible that UV-B exposure increases instability, resulting in further expansions that can lead to the *iil* phenotype. It has been previously shown that the transmission of UV-B on the epidermal surface of leaves is dependent on temperature with lower temperatures displaying reduced transmittance, due to the accumulation of the UV-B absorbing hydroxy cinnamic acid (Bilger et al., 2007). While it is possible to separate the UV-B and temperature effects under laboratory conditions, in the wild it is likely that plants will experience a combinatorial impact of both these factors. Thus, it is plausible that at elevated temperatures, there is also an impact of UV-B that leads to the observation of the *iil* phenotype. Taken together, our findings suggest that specific environmental changes unmask the *iil* phenotype in the Bur-0 accession of *Arabidopsis thaliana*.

### Epistatic interactions modify the phenotypic impact of the *IIL1* triplet repeat expansion in Col-0

We demonstrated that there are genetic modifiers present in Col-0 that suppress the *iil* phenotype. The *iil* phenotype does not segregate as a monogenic trait but it was difficult to discern whether it was digenic, simply based on the analysis of the phenotypes. We detected a strong epistatic interaction with a locus on chromosome 2. Epistatic interactions can either change the magnitude of the effect or can change the direction of effects (Mackay, 2014). A change in the magnitude of the effect can also be detected as an additive effect due to the second locus. Consistent with this, we also observe that there is an additive interaction detected between chromosome 2 and chromosome 4 for the *iil* phenotype. Therefore, it appears, while the repeat expansion determines whether the *iil* phenotype is conferred or not, its magnitude is altered by genetic modifiers. It appears that the modifier on chromosome 2 suppresses its phenotypic impact. In addition, the modifier on chromosome 5 also appears display a mild epistatic interaction that modifies the phenotype. We have confirmed at least the small effect QTL in chromosome 2, and the molecular isolation of this small effect QTL should reveal further interesting insights onto the underlying mechanisms for the *iil* phenotype.

### The *IIL1*GAA/TTC repeat expansion displays conditional neutrality

The *iil* phenotype is conditional and displayed only under specific environmental conditions. However, it has been previously shown that the loss of function of *IIL1* in the Col-0 background causes retarded growth even in standard growth conditions (Knill et al., 2009; Sureshkumar et al., 2009). Therefore, it is also possible that the *IIL1* repeat expansion could still reduce fitness. In this study, we have demonstrated that the *IIL1* triplet repeat expansions are recoverable from the wild even 50 years after the original collection of the Bur-0 accession. The recovery of the repeat expansion at multiple populations suggests that the *IIL1* repeat expansion is unlikely to be as detrimental as seen under our unusual lab conditions in natural habitats. The phenotypic consequences of this repeat expansion are possibly masked in the temperate Irish environment, in which temperatures of 27°C do not typically occur during short days.

QTL analysis for flowering time in the Bur-0 × Col-0 RILs revealed a major QTL mapping to the same region as the *IIL1* locus (not shown). This may simply be a reflection of the *iil* phenotype, which prevents the plants from reaching the flowering stage and not *per se* an effect of flowering. However, natural suppressors with reduced numbers of repeats in the Bur-0 background flowered earlier compared to Bur-0 in long day conditions (Figure S13). This suggests that the repeat expansion could delay flowering time. However, it is not yet clear what effect early flowering would have in the environment of the Burren, where the repeat expansion appears to be most prevalent. One possibility is that it could expose flowering plants to the risk of late frosts. Hence, we cannot exclude that a seemingly detrimental allele could provide an advantage in certain environments in the form of antagonistic pleiotropy, as described recently in the case of loss-of-function alleles for the *ICARUS1* gene (Zhu et al., 2015). The *IIL1* GAA/TTC polymorphism could thus be an ideal model for analyzing the underlying mechanisms and roles for such mutations in environmental adaptation.

### A New Island collection of genetically diverse local populations for evolutionary analysis

Our study has generated a new collection of genotyped strains from an island population that is suitable for studying natural variation in ruderal plants. We have also provided evidence that the Irish population displays both genotypic and phenotypic diversity, as other regional populations (Le Corre et al., 2002; Pico et al., 2008; Bomblies et al., 2010; Long et al., 2013; Brennan et al., 2014). Native to Eurasia and Northern Africa, *A. thaliana* is believed to have colonized Ireland in the post-glacial period (Francois et al., 2008), this Irish collection will also serve as an additional resource for genetic analysis into the history of the post-glacial colonization of Ireland by *A. thaliana*. The genetic diversity among the Irish population is extensive both in terms of genetic (SNP-based) diversity, and the diversity within the phenotypic traits analyzed. This notion is further supported by the measured phenotypic traits of flowering time, hypocotyl elongation, degree of leaf serration and germination all of which span response ranges similar to those observed between populations from other geographical regions.

### Variability of the non-expanded *IIL1* triplet repeats in Irish populations

The non-expanded GAA/TTC tracts in the Irish population spanned 3–28 repeats (Figure 3B), quite similar to that previously reported (0–36 repeats) across a global population set (Sureshkumar et al., 2009). Unusually, the Irish population exhibits a bimodal distribution in repeat lengths with peak frequencies of 9 and 26 repeats. This could indicate that the *IIL1* repeat in the Irish populations is comprised of two conserved genetic groups. We determined that certain repeat lengths were favored over others. For example, in our sequenced samples, 28 accessions had nine repeats, 14 had 11 repeats and only two had 10 repeats. It has been hypothesized that the number of repeats may reflect adaptations to the internal genetic environments (Undurraga et al., 2012). A previous study on polyglutamine (polyQ) tract variation within the *ELF3* gene (Undurraga et al., 2012) demonstrated that variation in coding repeats (e.g., polyQ) depend on genetic background, our results indicate the repeat lengths of non-coding trinucleotide repeats may also be confined in a background-dependent manner.

Overall, our study sheds light on the complex genetic interactions that modify the triplet repeat expansion associated *iil* phenotype. We have demonstrated the cryptic nature of this *iil* phenotype and conclude that even mutations with very strong phenotypes can be maintained in the wild if their effects are conditional. The *IIL1* triplet repeat expansion, which causes severe growth impairments at high ambient temperatures, has been maintained in the Irish populations for over 50 years. Though it is difficult to rule out balancing selection or fluctuating selection, our data suggests that, within Ireland, the allele might be neutral or beneficial. The Irish accessions of *A. thaliana*, along with the natural suppressors lines, provide an excellent resource to further analyze mechanisms that could explain the high frequency of the repeat expansion in the Irish populations. In addition, this genotyped collection of Irish strains will aid future studies to decipher the post-glacial colonization of the island of Ireland by *A. thaliana* accessions.

## Author contributions

Performed experiments (AT, SV, ES, and LC). Analyzed Date (AT, SV, AS, PM, TD, CD, ES, CS, and SB). Computational analysis of the data (AS, TD, and CD). Wrote the manuscript (AS, PM, CS, and SB). Designed and Supervised the study (CS and SB).

### Conflict of interest statement

The authors declare that the research was conducted in the absence of any commercial or financial relationships that could be construed as a potential conflict of interest.

## Supplementary material

Figure S1 **UV-B increases genetic instability of expanded repeats**. The region encompassing the *IIL1* repeat amplified through PCR is shown for several plants treated with UV-B compared to control plants.

Figure S2 **The visible comparative scale used for quantitatively scoring the *iil* phenotype. A-J** represents plants with increasing severity of the *iil* phenotype beginning with a normal plant (score of 1 in **A**) to the plant with the most severe *iil* phenotype (score of 10 in **J**). Each of the 2752 plants representing 344 RILs were scored against this scale to obtain a quantitative score for the *iil* phenotype and the average scores for each of the RILs was used in the QTL mapping reported in Figure 2.

Figure S3 **Variation observed among wild accessions at various collection sites in Ireland**. Arrows point to the plants. The leaves shown in J depict the variation observed between plants harboring serrated and non-serrated leaves in natural conditions.

Figure S4 **Recovery of accessions with the *IIL1* repeat expansion from Ireland. (A)** PCR analysis of few wild accessions displaying *IIL1* repeat expansion. **(B)** The *iil* phenotype recovered from the Irish accessions through a screen for the *iil* phenotype at 27°C. Scale bars = 1 cm.

Figure S5 **The Structure Harvester output that provides support for *K* = 2 populations in all Irish accessions. (A)** Graphical output of Evanno method as implemented in Structure Harvester for different *K*-values (from 1 to 8). **(B)** Quantitative output of the Evanno method as implemented in Structure harvester.

Figure S6 **Phylogenetic tree of genome-wide SNPs for all 131 samples**. Blue arrows show the accessions with the repeat expansions. Different colors highlight accessions Bur-0 (blue), Burren2011 (red), and Col-0 (green).

Figure S7 **Phylogenetic tree of based on SNPs in Chromosome 1**. Blue arrows show the accessions with the repeat expansions. Different colors highlight accessions Bur-0 (blue), Burren2011 (red), and Col-0 (green).

Figure S8 **Phylogenetic tree of based on SNPs in Chromosome 2**. Blue arrows show the accessions with the repeat expansions. Different colors highlight accessions Bur-0 (blue), Burren2011 (red), and Col-0 (green).

Figure S9 **Phylogenetic tree of based on SNPs in Chromosome 3**. Blue arrows show the accessions with the repeat expansions. Different colors highlight accessions Bur-0 (blue), Burren2011 (red), and Col-0 (green).

Figure S10 **Phylogenetic tree of based on SNPs in Chromosome 4**. Blue arrows show the accessions with the repeat expansions. Different colors highlight accessions Bur-0 (blue), Burren2011 (red), and Col-0 (green).

Figure S11 **Phylogenetic tree of based on SNPs in Chromosome 5**. Blue arrows show the accessions with the repeat expansions. Different colors highlight accessions Bur-0 (blue), Burren2011 (red), and Col-0 (green).

Figure S12 **The scale used for quantifying the levels of leaf serration in among the Irish accessions**.

Figure S13 **Repeat expansion has a minor effect on flowering time. (A,B)** Impact of repeat expansion on hypocotyl length at 27°C **(A)** and temperature sensitivity on hypocotyl elongation **(B)**. *p*-values refer to significance as determined through ANOVA. **(C)** QTL analysis at 27°C short days detects a minor effect QTL for flowering time spanning the repeat expansion (shown by the blue arrow). Please note that in this analysis, plants that displayed the *iil* phenotype are largely excluded as they do not reach the flowering stage **(D)** Loss of repeat expansion in Bur-0 leads to early flowering.

Table S1 **Primers used for analyzing and sequencing the repeat region of *IIL1***.

Table S2 **Genotypic and phenotypic data used for QTL analysis of the modifier of *IIL1***.

Table S3 **Details of the collection of accessions in Ireland**.

Table S4 **Phenotypic data used in this paper**. The accessions include the lines that are genotyped through DArT-seq.

Table S5 **Genotype results from 22 SSLP markers distributed throughout the genome**.

Table S6 **DArT-seq genotypic data of 4556 markers for the 131 lines**.

Table S7 **Mean phenotypic values and heritability for hypocotyl and root length in Irish accessions**.
