# Supplementary figures and images for: A Polynucleotide Repeat Expansion Causing Temperature-Sensitivity Persists in Wild Irish Accessions of Arabidopsis thaliana
Source: Front Plant Sci. 2016 Aug 31;7:1311. doi: 10.3389/fpls.2016.01311 (PMC5006647; doi:10.3389/fpls.2016.01311)

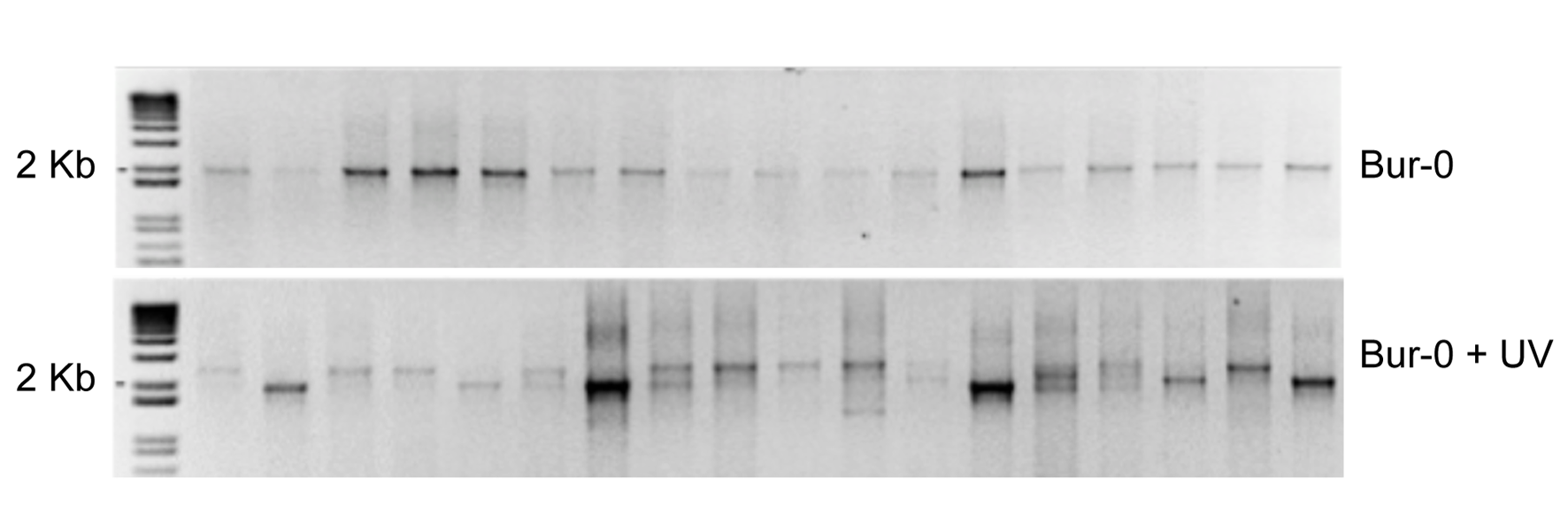

Supplement: Figure S1 — UV-B increases genetic instability of expanded repeats. The region encompassing the IIL1 repeat amplified through PCR is shown for several plants treated with UV-B compared to control plants. [file Image1.tif]

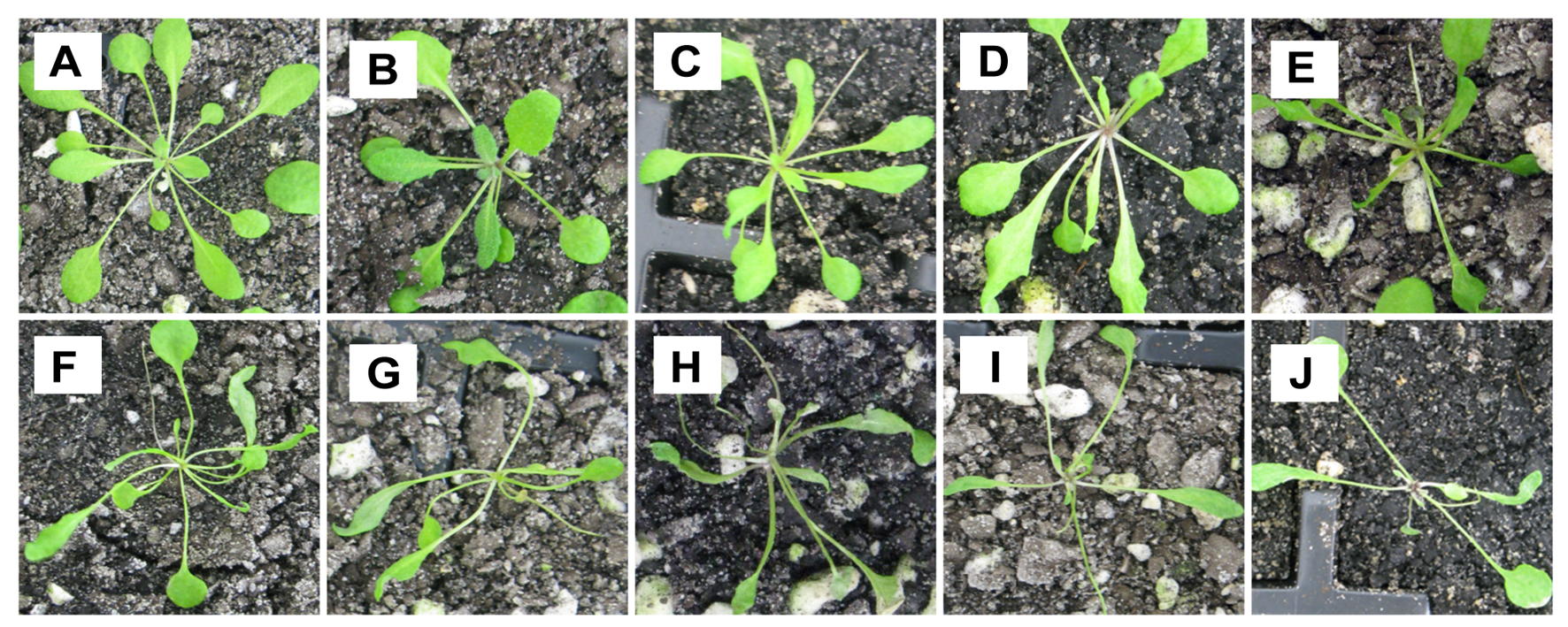

Supplement: Figure S2 — The visible comparative scale used for quantitatively scoring the iil phenotype. A-J represents plants with increasing severity of the iil phenotype beginning with a normal plant (score of 1 in A) to the plant with the most severe iil phenotype (score of 10 in J). Each of the 2752 plants representing 344 RILs were scored against this scale to obtain a quantitative score for the iil phenotype and the average scores for each of the RILs was used in the QTL mapping reported in Figure 2. [file Image2.tif]

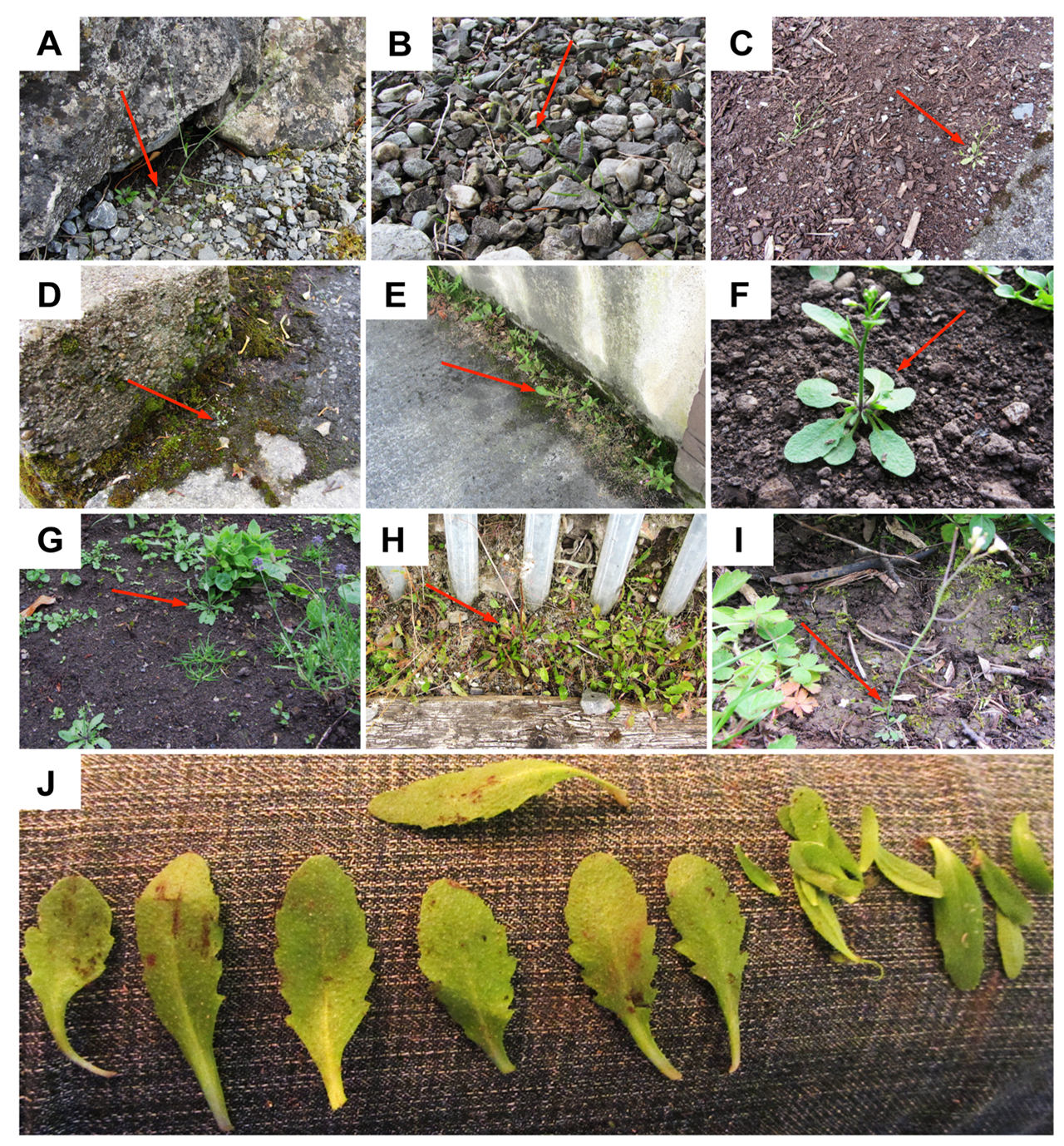

Supplement: Figure S3 — Variation observed among wild accessions at various collection sites in Ireland. Arrows point to the plants. The leaves shown in J depict the variation observed between plants harboring serrated and non-serrated leaves in natural conditions. [file Image3.tif]

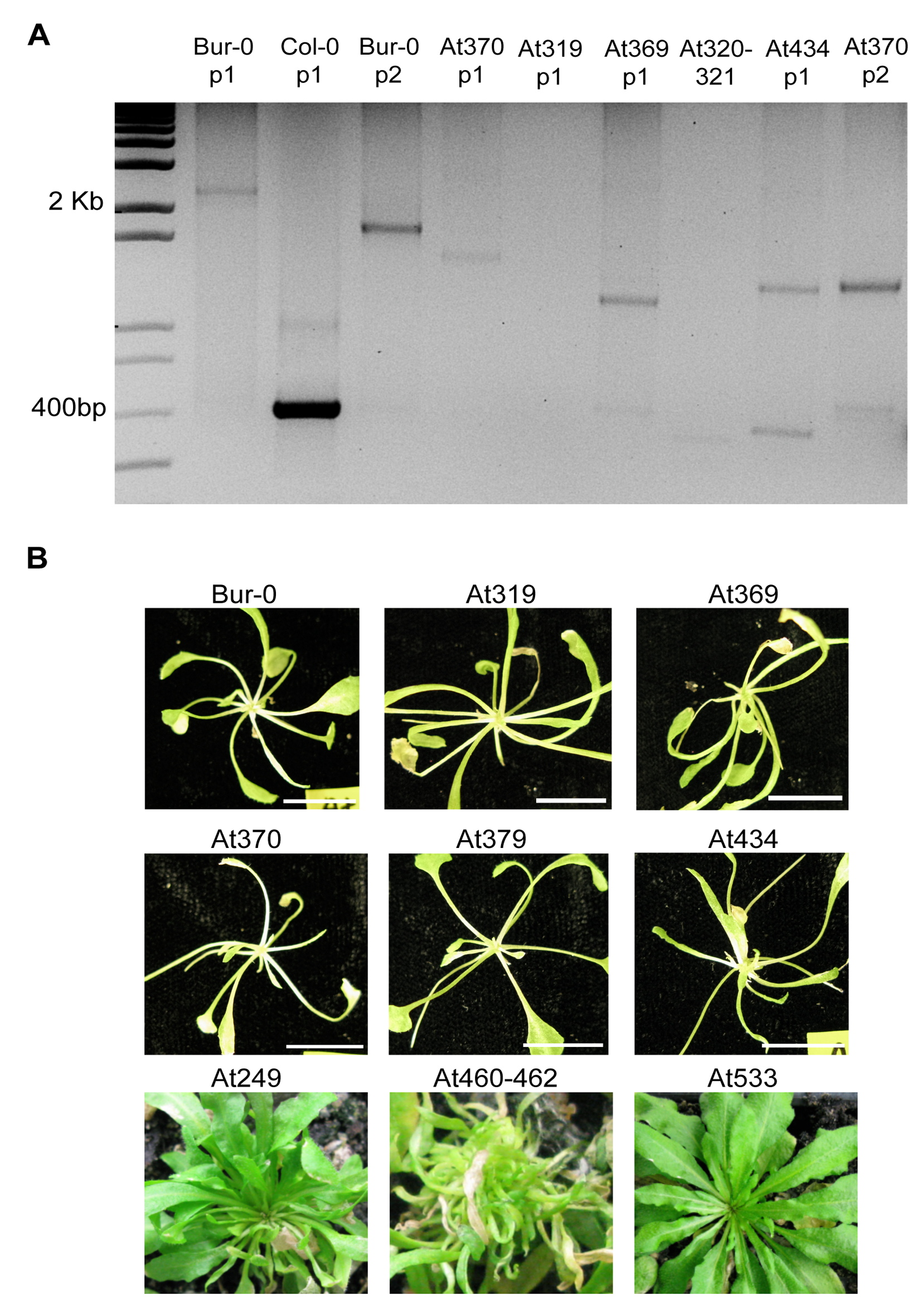

Supplement: Figure S4 — Recovery of accessions with the IIL1 repeat expansion from Ireland. (A) PCR analysis of few wild accessions displaying IIL1 repeat expansion. (B) The iil phenotype recovered from the Irish accessions through a screen for the iil phenotype at 27°C. Scale bars = 1 cm. [file Image4.tif]

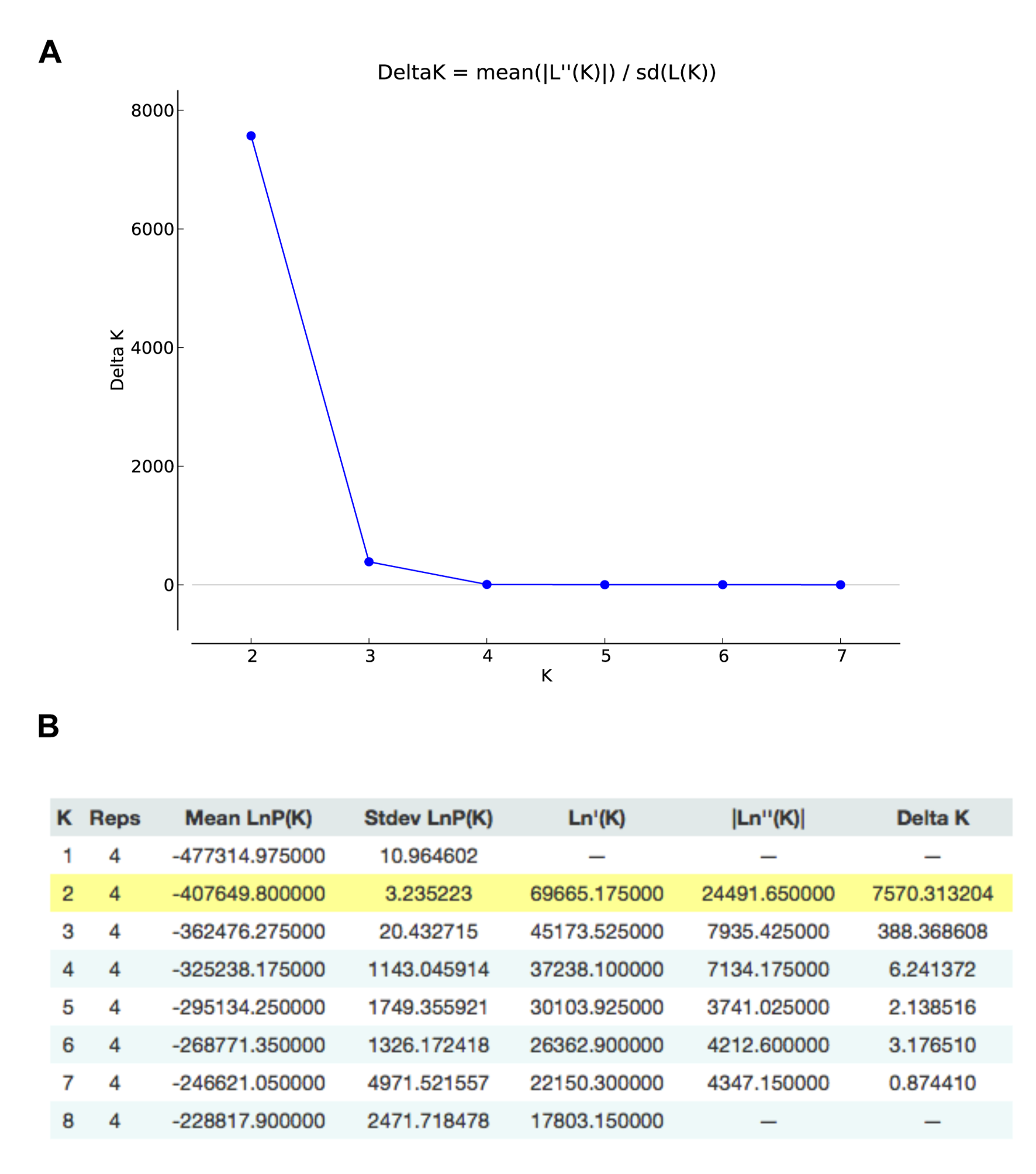

Supplement: Figure S5 — The Structure Harvester output that provides support for K = 2 populations in all Irish accessions. (A) Graphical output of Evanno method as implemented in Structure Harvester for different K-values (from 1 to 8). (B) Quantitative output of the Evanno method as implemented in Structure harvester. [file Image5.tif]

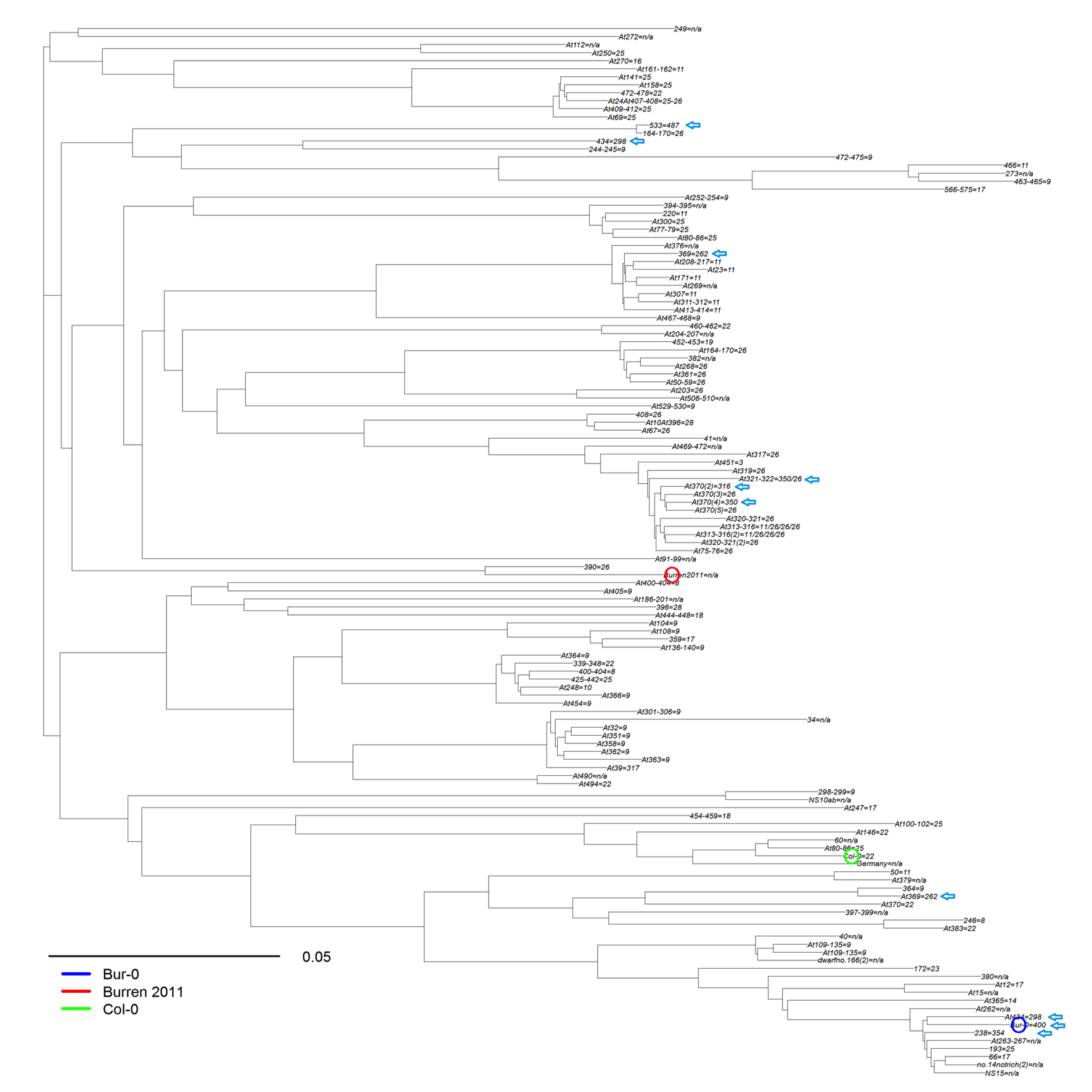

Supplement: Figure S6 — Phylogenetic tree of genome-wide SNPs for all 131 samples. Blue arrows show the accessions with the repeat expansions. Different colors highlight accessions Bur-0 (blue), Burren2011 (red), and Col-0 (green). [file Image6.tif]

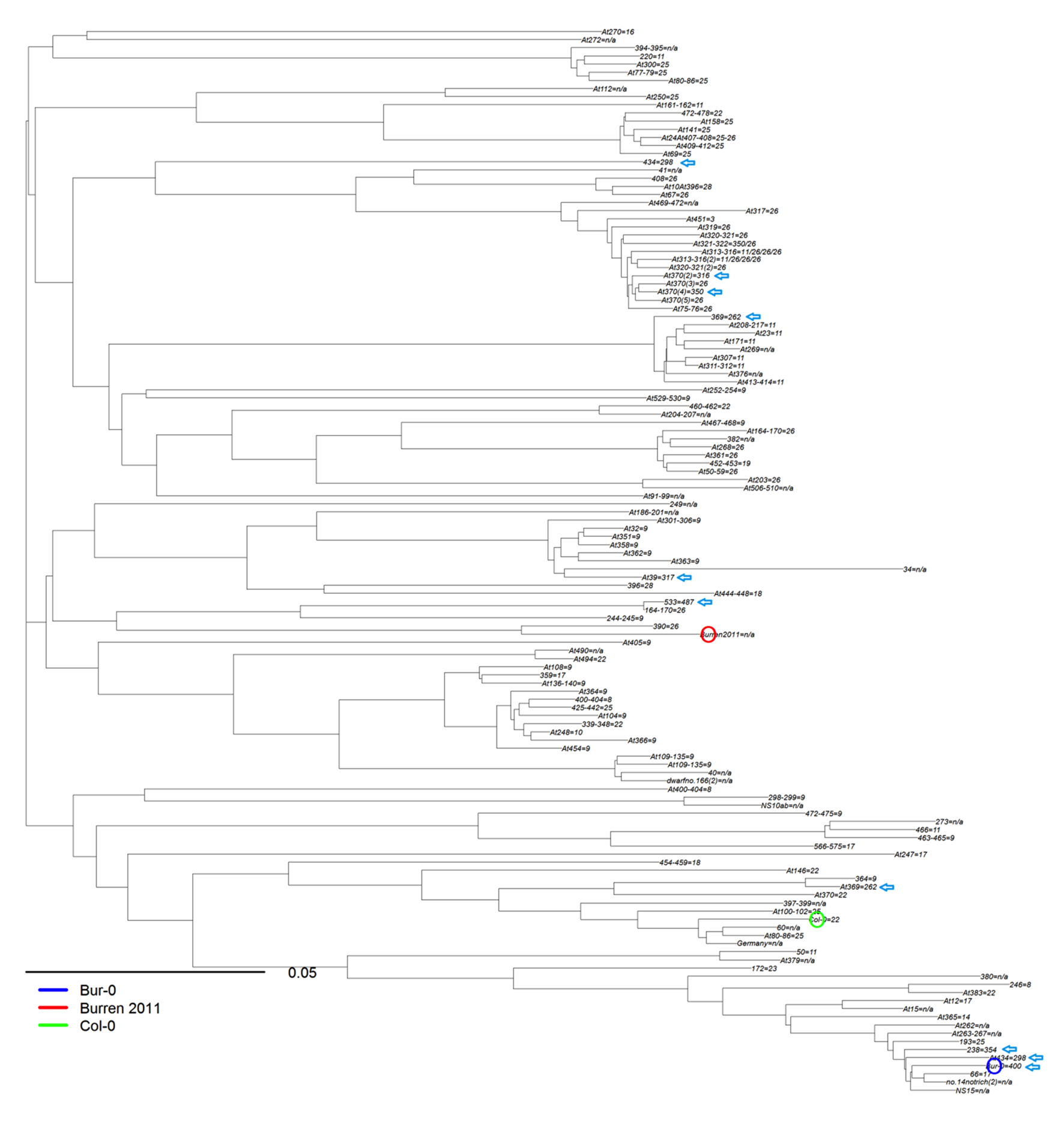

Supplement: Figure S7 — Phylogenetic tree of based on SNPs in Chromosome 1. Blue arrows show the accessions with the repeat expansions. Different colors highlight accessions Bur-0 (blue), Burren2011 (red), and Col-0 (green). [file Image7.tif]

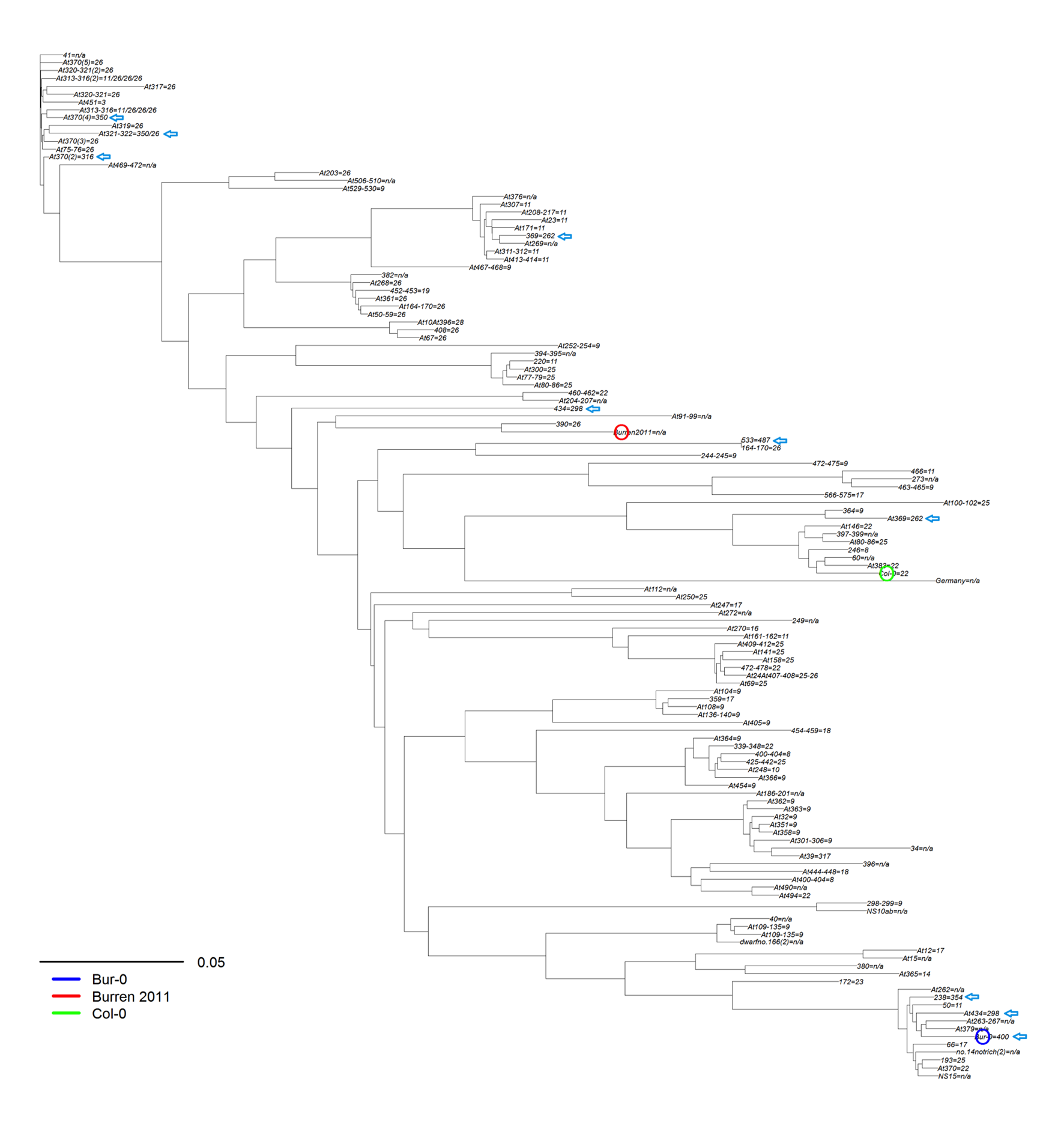

Supplement: Figure S8 — Phylogenetic tree of based on SNPs in Chromosome 2. Blue arrows show the accessions with the repeat expansions. Different colors highlight accessions Bur-0 (blue), Burren2011 (red), and Col-0 (green). [file Image8.tif]

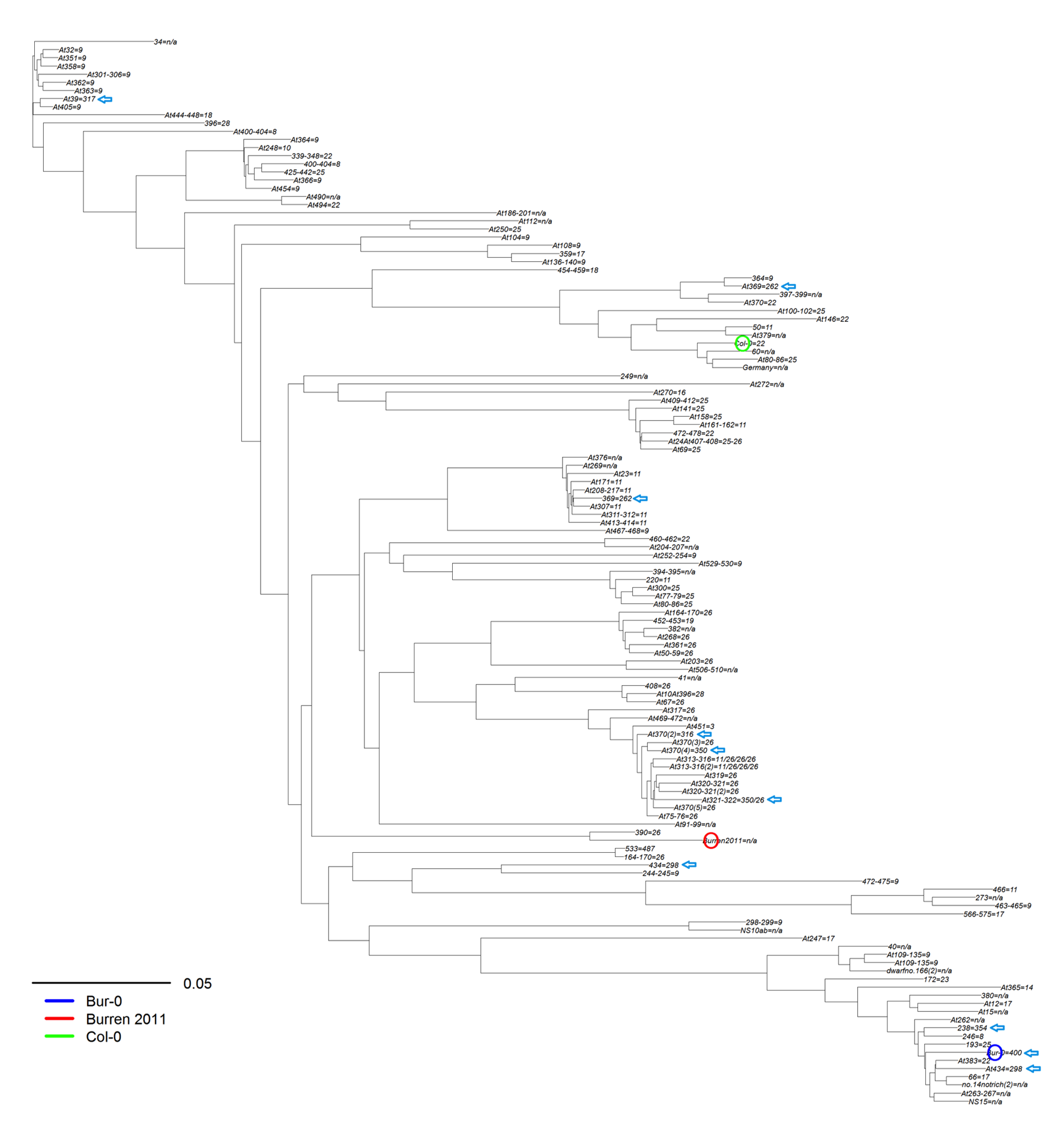

Supplement: Figure S9 — Phylogenetic tree of based on SNPs in Chromosome 3. Blue arrows show the accessions with the repeat expansions. Different colors highlight accessions Bur-0 (blue), Burren2011 (red), and Col-0 (green). [file Image9.tif]

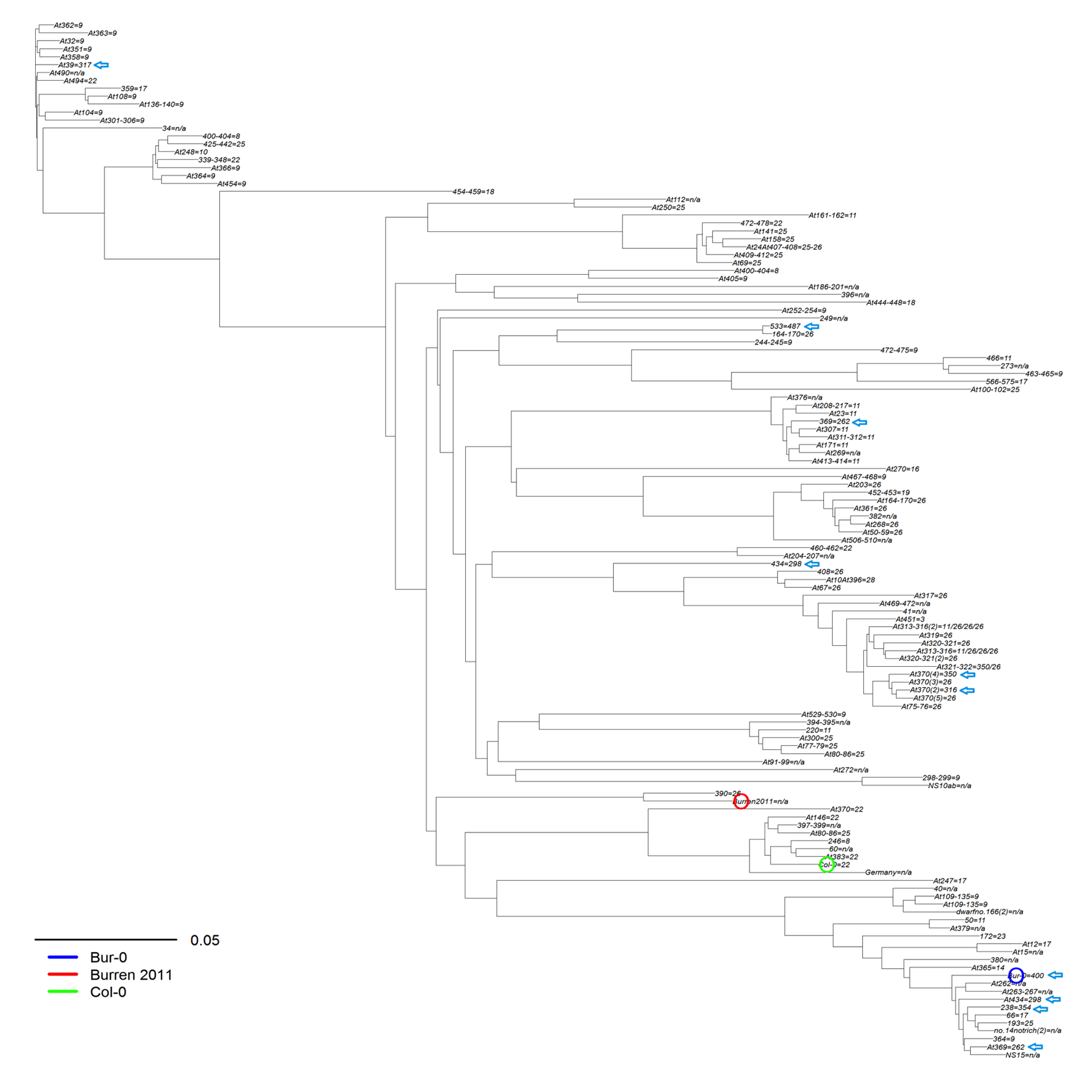

Supplement: Figure S10 — Phylogenetic tree of based on SNPs in Chromosome 4. Blue arrows show the accessions with the repeat expansions. Different colors highlight accessions Bur-0 (blue), Burren2011 (red), and Col-0 (green). [file Image10.tif]

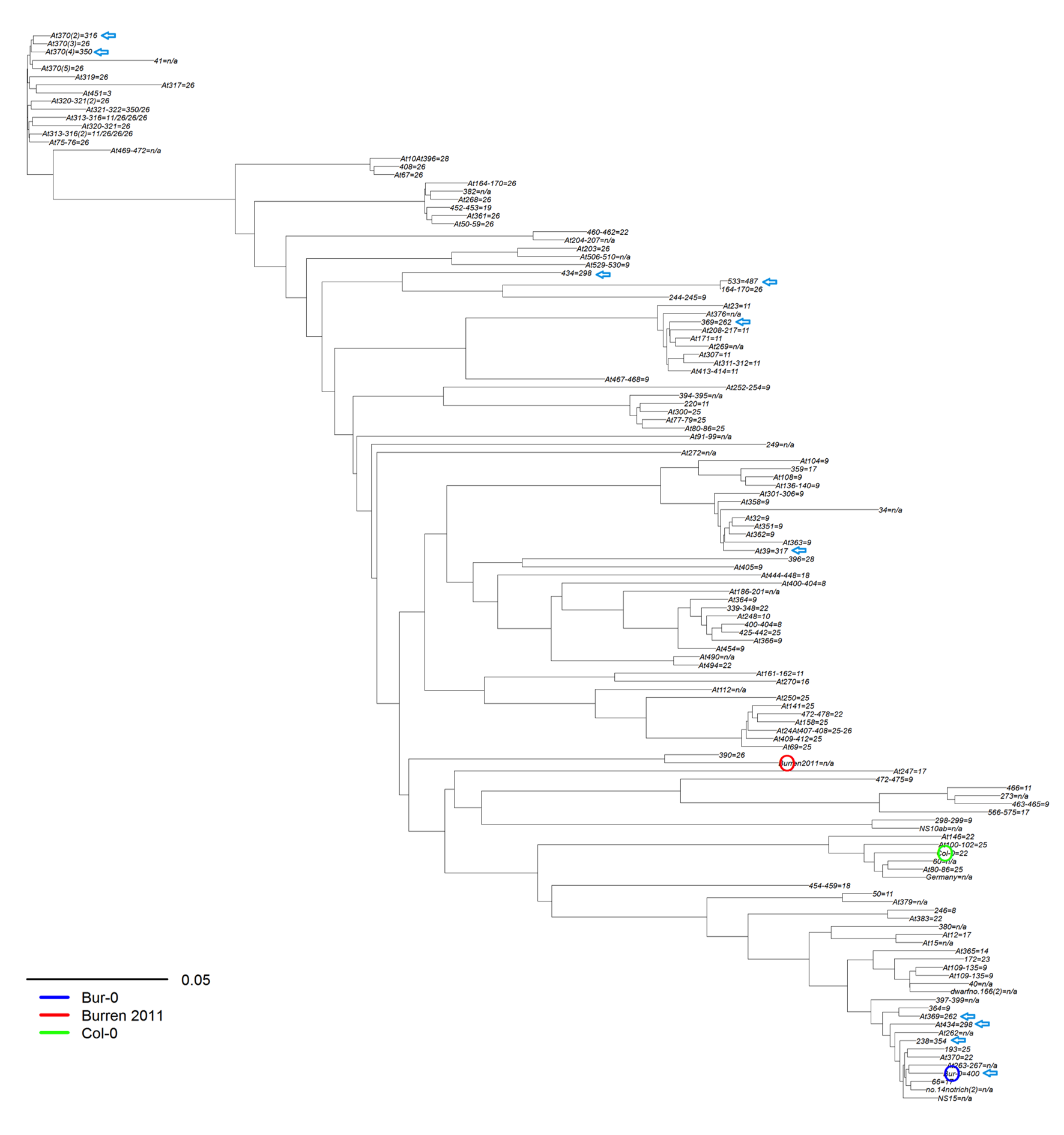

Supplement: Figure S11 — Phylogenetic tree of based on SNPs in Chromosome 5. Blue arrows show the accessions with the repeat expansions. Different colors highlight accessions Bur-0 (blue), Burren2011 (red), and Col-0 (green). [file Image11.tif]

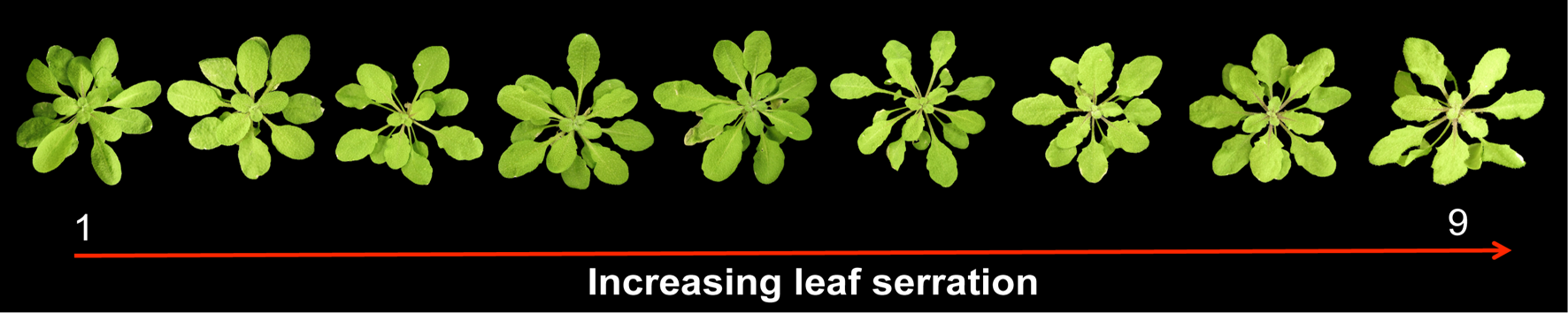

Supplement: Figure S12 — The scale used for quantifying the levels of leaf serration in among the Irish accessions. [file Image12.tif]

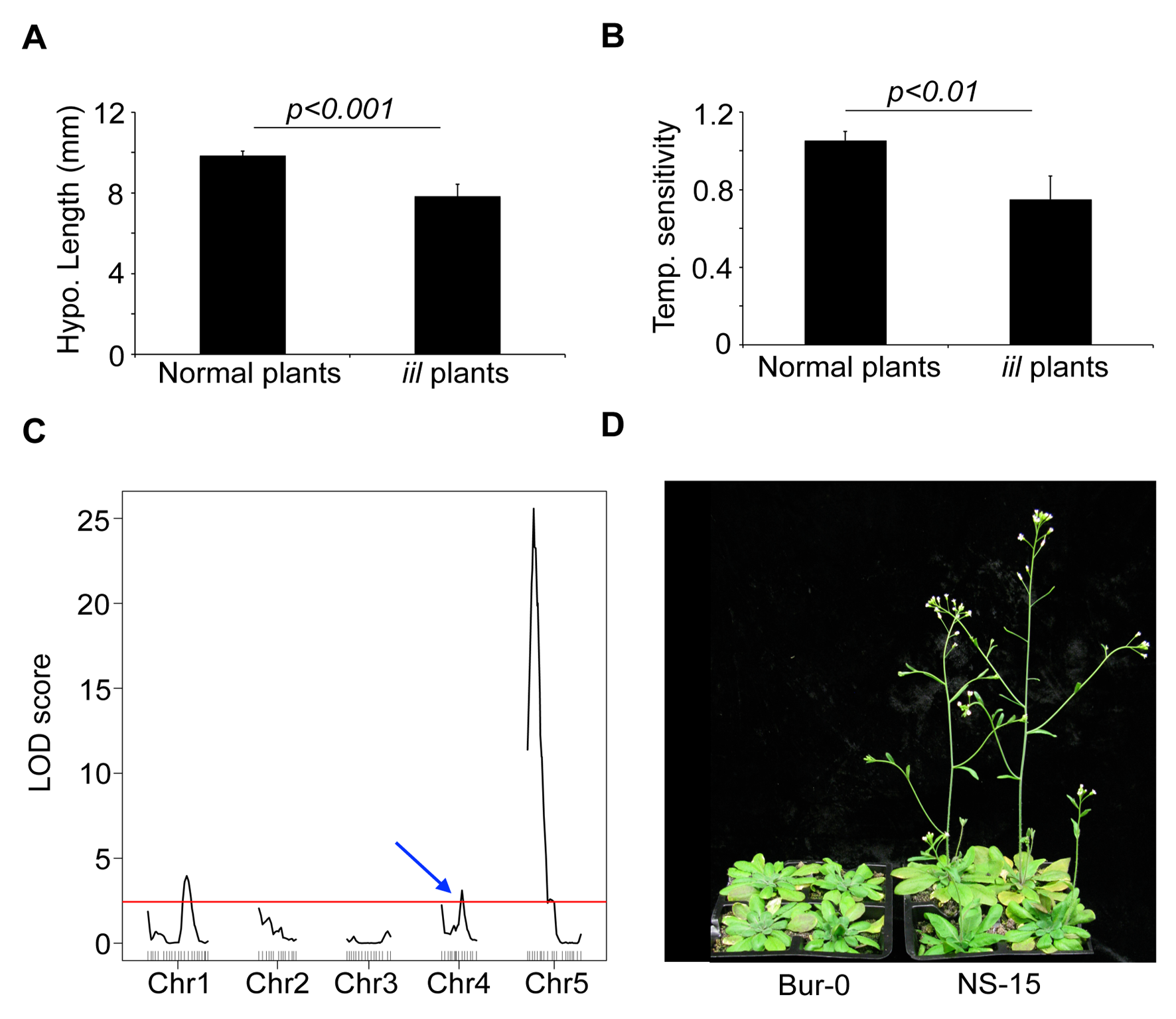

Supplement: Figure S13 — Repeat expansion has a minor effect on flowering time. (A,B) Impact of repeat expansion on hypocotyl length at 27°C (A) and temperature sensitivity on hypocotyl elongation (B). p-values refer to significance as determined through ANOVA. (C) QTL analysis at 27°C short days detects a minor effect QTL for flowering time spanning the repeat expansion (shown by the blue arrow). Please note that in this analysis, plants that displayed the iil phenotype are largely excluded as they do not reach the flowering stage (D) Loss of repeat expansion in Bur-0 leads to early flowering. [file Image13.tif]
